# Outcomes of Left Main Revascularization after Percutaneous Intervention or Bypass Surgery

**DOI:** 10.1155/2022/6496777

**Published:** 2022-04-12

**Authors:** Fernando Scudiero, Iacopo Muraca, Angela Migliorini, Rossella Marcucci, Matteo Pennesi, Lapo Mazzolai, Nazario Carrabba, Niccolò Marchionni, Pierluigi Stefano, Renato Valenti

**Affiliations:** ^1^Medical Sciences Departement, Cardiology Unit, ASST Bergamo Est, Bolognini Hospital, Seriate, Bergamo, Italy; ^2^Division of Interventional Cardiology, Cardiothoracovascular Department, Careggi University Hospital, Florence, Italy; ^3^Department of Clinical and Experimental Medicine, University of Florence, Florence, Italy; ^4^Division of Cardiac Surgery Unit, Cardiothoracovascular Department, Careggi University Hospital, Florence, Italy

## Abstract

**Background:**

This study is aimed at comparing the clinical outcomes of unprotected left main coronary artery disease (ULMCAD) treatment with contemporary percutaneous coronary intervention (PCI) or coronary artery bypass grafting (CABG) in a “real-world” population.

**Methods and Results:**

Overall, 558 consecutive patients with ULMCAD (mean age 71 ± 9 years, male gender 81%) undergoing PCI or CABG were compared. The primary endpoint was the composite of death, nonfatal myocardial infarction, or stroke. Diabetes was present in 29% and acute coronary syndrome in 56%; mean EuroSCORE was 11 ± 8. High coronary complexity (SYNTAX score >32) was present in 50% of patients. The primary composite endpoint was similar after PCI and CABG up to 4 years (15.5 ± 3.1% vs. 17.1 ± 2.6%; *p*=0.585). The primary end point was also comparable in a two propensity score matched cohorts. Ischemia-driven revascularization was more frequently needed in PCI than in CABG (5.5% vs. 1.5%; *p*=0.010). By multivariate analysis, diabetes mellitus (HR 2.00; *p*=0.003) and EuroSCORE (HR 3.71; *p* < 0.001) were the only independent predictors associated with long-term outcome.

**Conclusions:**

In a “real-world” population with ULMCAD, a contemporary revascularization strategy by PCI or CABG showed similar long-term clinical outcome regardless of the coronary complexity.

## 1. Introduction

Unprotected left main coronary artery disease (ULMCAD) is associated with increased risk of serious adverse events due to the large amount of myocardium at risk. Historically, coronary artery bypass grafting (CABG) was recommended as the revascularization strategy of choice for ULMCAD [1, 2]. Nevertheless, the role of percutaneous coronary intervention (PCI) for the treatment of ULMCAD has rapidly gained importance during the past decade, driven by the technological advances of drug-eluting stents (DESs), antithrombotic therapy, procedural strategies, and interventional cardiologists expertise [3, 4].

Large registries [5, 6] and randomized clinical trials (RCTs) [7–11] reported favorable outcomes of PCI in ULMCAD; consequently, current guidelines support ULMCAD PCI as a feasible alternative to CABG in selected patients [12, 13]. Nonetheless, concerns about the optimal revascularization strategy for ULMCAD were raised by the long-term conflicting results of the largest and most recent studies [14, 15] (the Evaluation of XIENCE versus Coronary Artery Bypass Surgery for Effectiveness of Left Main Revascularization (EXCEL) trial and the Nordic-Baltic-British left main revascularization (NOBLE) trial), which are endorsed by the updated European Society of Cardiology guidelines on myocardial revascularization [13]. Furthermore, clinical outcomes of ULMCAD revascularization, either by PCI or CABG, in “real world” settings are still debated.

This study aims to compare the clinical outcomes of ULMCAD patients treated with either PCI or CABG accomplished by contemporary technical and global clinical strategies, in a “real-world” population managed in a high-volume referral center.

## 2. Methods

### 2.1. Study Population

This study includes all consecutive patients who underwent revascularization for ULMCAD between 2013 and 2016 in our high-volume (PCI procedures >1500 per year and CABG interventions >500 per year) referral center. ULMCAD was defined as a de novo ≥50% stenosis of left main coronary artery at selective angiography. Patients with stable coronary artery disease, as well those with acute coronary syndromes, were included irrespectively of their coronary anatomy. All angiograms were scored according to the SYNTAX algorithm [16].

The clinical decision making process and the revascularization strategy choice were endorsed/shared by interventional cardiologist, cardiac surgeon, and referral cardiologist following the model of a “minimalist” Heart Team [17, 18] in patients with stable coronary artery disease and/or nonemergent/urgent indications. The choice of the revascularization strategies pointed at achieving the most complete revascularization in any patient. Logistic EuroSCORE was calculated for each patient; high surgical risk was defined as a EuroSCORE ≥6 [19]. All PCI patients received 2^nd^ generation DES. For distal left main disease, a single-stent technique was preferred in patients with a normal or diminutive appearing side branch, whereas a double-stent technique was considered in patients with disease of both ostia and proximal segments of left anterior descending and circumflex arteries. Regardless of the stenting technique used, routine final kissing balloon inflation and proximal optimization technique with noncompliant balloons was performed. Intravascular ultrasound guidance was strongly recommended.

Multivessel disease was defined as stenosis >70% of >1 major coronary artery at baseline angiography. Anatomical-based definition of complete coronary revascularization was performed on post-PCI angiography evaluation as a TIMI flow grade 3 with residual stenosis <30% on visual assessment in the three main coronary arteries and their branches >2 mm of diameter achieved either during the index hospitalization or at any time within 30 days after ULMCAD PCI.

Chronic antithrombotic treatment included aspirin (100 mg/day, indefinitely) and ticagrelor 90 mg BID or prasugrel (5 or 10 mg daily as appropriate), or clopidogrel 75 mg daily for at least 6 months. Long-term DAPT (>12 months) was strongly recommended [20]. Patients with ACS and/or extended coronary disease received second-generation P2Y12 inhibitor antiplatelet agent [12].

According to our institutional protocol, all patients treated with clopidogrel, platelet reactivity was assessed by a light transmission aggregometry laboratory (LTA) test (APACT4, Helena Laboratories, Milan, Italy); high on-treatment platelet reactivity on treatment (HTPR) was defined as residual platelet aggregation by 10 *μ*mol ADP ≥70% [21–23]. All patients resulting nonresponders to clopidogrel were escalated to prasugrel or ticagrelor. Other drugs such as betablockers, angiotensin-converting enzyme inhibitors, and statins were used in accordance with recommended practice [12]. Unscheduled angiography was allowed based on clinical indication.

CABG was performed using standard techniques to achieve a complete anatomical-based revascularization as defined for the PCI group. The use of off-pump technique and bilateral internal mammary arteries (BIMA) grafts were strongly recommended whenever possible. Intraoperative, transesophageal echocardiography was recommended to assess the ascending aorta and the ventricular and valvular function. In all patients aspirin was administered during the perioperative period. In patients undergoing CABG, antithrombotic therapy was administered according to current guidelines [24].

The study was approved by the institutional ethics committee and complies with the Declaration of Helsinki. Informed consent has been obtained from all subjects or their caregivers.

### 2.2. Endpoints

The primary study endpoint was a composite of all-cause death, nonfatal MI, or stroke at 4 years. All other endpoints (the individual components of the composite endpoint, cardiac death and ischemia-driven revascularization) were considered as explorative. Cardiac death included death resulting from MI, heart failure, sudden cardiac death, and death due to cardiac procedures. Spontaneous MI was defined as the occurrence >72 hours after any PCI or CABG of the rise and/or fall of cardiac biomarkers >1x URL*∗* plus ECG changes indicative of new ischemia, or development of pathological *Q* waves, or angiographically documented graft or native coronary artery occlusion or new severe stenosis with thrombosis and/or diminished epicardial flow, or imaging evidence of new loss of viable myocardium or new regional wall motion abnormality [25]. Stroke was defined as an acute neurological defect lasting more than 24 hours. All revascularization management after index procedure were driven by occurrence of symptoms and/or ischemia.

### 2.3. Follow-Up

All patients had scheduled follow-up visits at 1, 6, and 12 months and annually thereafter. All patients were contacted by telephone interviews to obtain 4-year follow-up. All other possible information gathered from hospital readmission charts or by referring physicians, relatives, or municipality vital registries, were entered into the prospective database.

### 2.4. Statistical Analysis

Categorical data are expressed as frequencies and continuous data as mean ± SD or median and interquartile range for normal and non-normal distributions, respectively. The *χ* test was used to compare categorical variables, and the unpaired two-tailed Student's *t*-test or Mann–Whitney rank-sum test to analyze differences between continuous variables. Cumulative incidence curves were generated following the Kaplan–Meier method, assessing between groups differences with the log-rank test. Multivariable regression analysis to evaluate the independent contribution of clinical, angiographic, procedural variables to the primary endpoint was performed by Cox proportional hazards model. The variables entered into the model were revascularization strategy (PCI or CABG), SYNTAX score >32, diabetes mellitus, complete revascularization and EuroSCORE ≥13. The risk of overfitting was controlled by using a ratio of at least 1 : 100 for the number of variables and sample size. The proportional hazard assumption was assessed and satisfied graphically by plotting log (−log) survival curves against log survival time for each predictor category and verifying whether curves were parallel. We performed sensitivity analysis in order to test how robust the model was relative to the included population by assessing the effect of excluding STEMI patients.

A Cox proportional hazards model was also used to test interactions. In order to minimize the bias due to the nonrandomized nature of the study and the possibility of overfitting, a propensity score analysis was performed with a logistic regression model from which the probabilities for the type of revascularization (PCI or CABG) was calculated for each patient. The variables entered into the model were age, male gender, diabetes mellitus, ACS, left ventricular ejection fraction (LVEF) <40%, three-vessel disease, right coronary artery chronic total occlusion, SYNTAX score >32, and EuroSCORE. Model discrimination was assessed with the C statistic and goodness of fit with the Hosmer–Lemeshow test. A Cox multivariable analysis was then performed using the propensity score as a continuous covariate.

Matched analysis was also performed because of expected differences between PCI and CABG revascularization groups. An optimal data matching technique (1 : 1) was performed with a random order using the greedy-matching algorithm for propensity score difference and forcing the imbalanced characteristics (SYNTAX score >32, LVEF <40%, diabetes and EuroSCORE >13). Bias reduction was assessed by comparing the standardized difference before and after matching between the 2 groups (a value <10% after matching indicates inconsequential imbalance). After matching, the standardized difference changed from 67% to 9%. Four-year outcomes for the primary endpoint were assessed after matching by Kaplan–Meier analysis. Hazards ratio (HR) and their 95% confidence intervals (CI) were calculated. All tests were two-tailed. A *p* value <0.05 was considered significant. Analyses were performed with SPSS statistical package, version 21 (IBM Corp., Armonk, NY, USA).

## 3. Results

### 3.1. Patient Population and Procedural Outcome

Overall, 558 consecutive patients who underwent revascularization for ULMCAD (52% vs. 48%, respectively in PCI and CABG groups) were included in the present analysis. Main baseline characteristics are summarized in Table 1. The rates of ST-elevation myocardial infarction (10% vs. 2%; *p* < 0.001, respectively, in PCI and CABG groups) and left ventricular dysfunction (28% vs. 14%; *p* < 0.001) were higher in the PCI group. Female patients were 107 (19%). Of them, 60 (20%) were treated with PCI, while 47 (17%) underwent CABG. Most of patients (84%) in PCI group were on DAPT with ticagrelor or prasugrel, while 34 (12%) were treated with clopidogrel; of them, 13 patients resulted HTPR and were escalated to a more potent P2Y12 inhibitor.

Main angiographic and procedural characteristics are summarized in Table 2.

In the overall population, high-quality procedural standards were guaranteed by adherence to contemporary, guidelines-based strategies: all PCI patients received everolimus-eluting stents 2nd generation DES for left main stenting; rate of IVUS-guided stenting was 74%. Off-pump procedure and BIMA graft were adopted, respectively, in 81% and 58% of the CABG cohort. A complete revascularization was accomplished in 83% of the overall population and in 86% vs. 81% of the CABG and PCI group, respectively (*p*=0.086). Conversely, the PCI group had a much shorter mean hospital stay (4 ± 3 vs. 10 ± 5 days; *p* < 0.001).

### 3.2. Clinical Outcome

**Figure 1:**
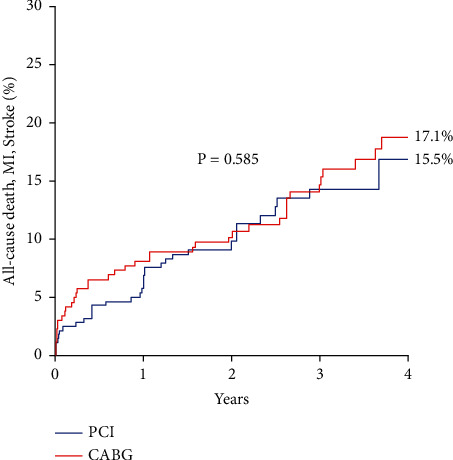
Kaplan–Meier curves for composite primary endpoint according to revascularization strategy by PCI or CABG (overall study population). MI, myocardial infarction; PCI, percutaneous coronary intervention; CABG, coronary artery bypass grafting.

**Figure 2:**
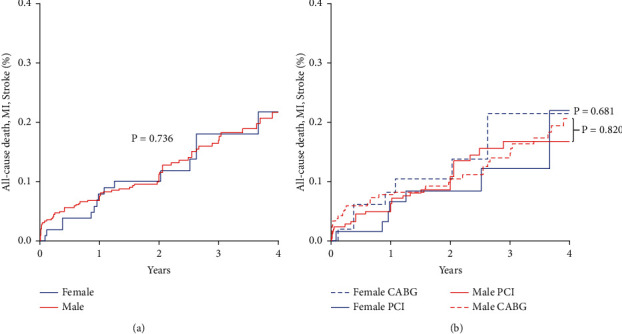
(a) Kaplan–Meier curves for composite primary endpoint according to gender. MI, myocardial infarction; PCI, percutaneous coronary intervention; CABG, coronary artery bypass grafting. (b) Kaplan–Meier curves for composite primary endpoint according to gender and strategy of revascularization. MI, myocardial infarction; PCI, percutaneous coronary intervention; CABG, coronary artery bypass grafting.

The median follow-up length was 3 years (IQR 2–4 years). The cumulative incidence of the primary composite endpoint was similar in PCI and CABG groups up to 4 years: 15.5 ± 3.1% vs. 17.1 ± 2.6%, respectively, *p*=0.585 (Figure 1). Similar results in PCI and CABG groups were found also for patients with high coronary complexity (left main and three-vessel disease): 15.3 ± 3.4% vs. 17.8 ± 3.4%; *p*=0.687, respectively. No significant difference was found in overall mortality in the two groups (11.1 ± 2.1% vs. 15.2 ± 2.5%; *p*=0.443). Other explorative endpoints are reported in Table 3. Ischemia-driven revascularization was low in overall study population (8%), but significantly more frequent in PCI than in CABG cohort (6% vs. 2%; *p*=0.010). As depicted in Figure 2 panel A, female gender did not impact the incidence of the primary endpoint at 4 years (*p*=0.736); equally, the revascularization strategy by PCI or CABG did not influence the outcome either in male or in female patients (Figure 2(b)).

**Figure 3:**
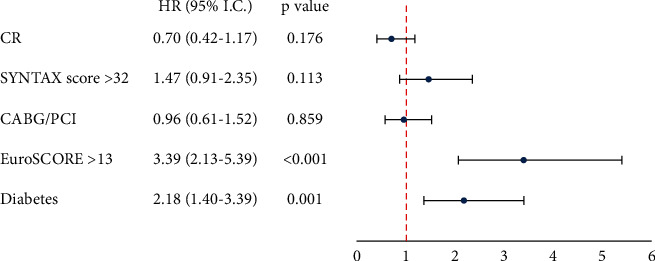
Multivariate analysis for the composite of death, nonfatal myocardial infarction, or stroke. CABG, coronary artery bypass grafting; CR, complete revascularization; PCI, percutaneous coronary intervention.

At multivariable analysis, revascularization strategy by PCI or CABG was not independently associated with the composite primary endpoint, which was associated with diabetes mellitus and EuroSCORE (Figure 3) even after propensity score adjustment (HR 2.00; 95% CI: 1.27 to 3.18; (*p*=0.003) and HR 3.71; 95% CI 2.35 to 5.85; (*p* < 0.001), respectively (C statistic = 0.63, *p* < 0.001; *p*=0.478 for Hosmer–Lemeshow test)). Furthermore, the interactions between revascularization strategy vs. EuroSCORE >13 (*p*=0.605) and revascularization strategy vs. SYNTAX score >32 (*p*=0.112) did not resulted significant.

Also, in sensitivity analysis with exclusion of STEMI patients, revascularization strategy by PCI or CABG was not independently associated with the composite primary endpoint (HR 0.882; 95% CI: 0.55 to 1.41; (*p*=0.599)).

After propensity score matching (1 : 1), we identified 404 patients with balanced baseline characteristics that are summarized in Table 4. In the matched population, the composite primary endpoint up to 4 years (13.1 ± 2.7% vs. 14.4 ± 2.9%; *p*=0.773) and all-cause death (10.4 ± 2.4% vs. 13.4 ± 2.8%; *p*=0.995) were similar in PCI and CABG groups, respectively. Similar results between PCI and CABG groups were found also in patients with high coronary complexity (left main and three-vessel disease): 17.2 ± 4.0% vs. 16.1 ± 3.9% *p*=0.634, in PCI and CABG groups, respectively.

## 4. Discussion

The main findings of this “real world” registry, involving patients with ULMCAD treated with PCI or CABG, are as follows: (1) long-term primary composite endpoint of death, nonfatal MI, or stroke are comparable in PCI and CABG groups; (2) EUROScore and diabetes were the only variables independently associated with the composite clinical outcome; (3) the primary endpoint was independent of coronary complexity evaluated by SYNTAX score and revascularization strategy; and (4) female gender did not impact the clinical outcome, regardless of revascularization strategies by PCI or CABG.

In the last decades, several studies and meta-analyses enrolling patients with ULMCAD showed comparable long-term outcomes, irrespective of revascularization strategy; nonetheless, patients undergoing PCI have an increased risk of target vessel revascularization [8, 9, 26–30].

The results of our study support that contemporary revascularization strategy by PCI or CABG in a “real-world” population are comparable in patients with ULMCAD, regardless of the presence of three-vessel disease and/or high complexity coronary anatomy.

Our findings expand the results of recent RCTs to a “real-world,” unselected population characterized by a great burden of comorbidities, a high anatomic coronary complexity, and on average, a more critical clinical presentation compared to the selected population enrolled in trials. Indeed, patients enrolled in EXCEL and NOBLE trials were younger (median age: 66 years) and had a lower rate of ACS (39% and 18%, respectively) and less comorbidities (mean EuroSCORE was 2%) than our study population. Remarkably, in our study population, the rate of patients with high coronary complexity (SYNTAX score >32) was 50%, whereas in the NOBLE trial was 8% and in EXCEL trial such patients were excluded by study protocol (although a posthoc corelab analysis showed a 25% of SYNTAX score >32) [31].

The favorable clinical outcomes in our study can be explained by the strict adoption of contemporary revascularization strategies for both PCI and CABG, including an updated antithrombotic therapy, the most complete revascularization possible, use of second-generation DES, high rate of intracoronary imaging in the PCI group and off-pump technique, and arterial grafts and BIMA in the CABG group. Conversely, some characteristics of the abovementioned RCTs [10, 11] might have affected the endpoints of the studies. In the NOBLE trial, 10% of patients received first-generation DES and all patients, including those with ACS at presentation, received aspirin and clopidogrel rather than the newest P2Y12 inhibitors. Similarly, in the EXCEL trial, 73% of patients undergoing PCI received aspirin and clopidogrel, including those with ACS. Therefore, the beneficial effect of new antiplatelet P2Y12 inhibitors was negligible. Moreover, the HTPR was never investigated in RCTs. A complete revascularization although in the NOBLE trial was achieved in 543 (92%) of 592 patients treated with PCI, in the CABG group of the EXCEL trial reached only 24.8%. Finally in EXCEL trial, all-cause death occurred more frequently in the PCI arm compared with the CABG arm (13% versus 9.9%); however, 58 of 119 deaths due to any cause in the PCI arm were adjudicated as noncardiovascular deaths.

Other large registries [6, 30] have previously compared long-term clinical outcomes between PCI and CABG. In the multicenter DELTA 2 registry [32], 3,986 patients with LMCAD treated by PCI with second-generation DES were compared with those from the historical DELTA 1 CABG cohort [33], using a propensity score matching technique at a median follow-up of 17 months, and the primary endpoint (a composite of death, MI, or cerebrovascular accident) occurred in 10.4% of patients who underwent PCI, a finding consistent with our results.

Conversely, the results of a single-center study of Zheng et al. [6], conducted in 4,046 patients between 2004 and 2010, demonstrated that CABG was associated with improved outcomes at 3 years, especially in patients with more complex disease. The 10-year follow-up in the MAIN-COMPARE registry showed a better clinical outcome with CABG, but contemporary strategies were not used in PCI revascularization [34]. Notably, the results of all these registries were extrapolated from cohorts of patients younger, with a lower risk EuroSCORE and a less complex coronary anatomy than those in our study; furthermore, complete coronary revascularization rate was very low or not reported. Therefore, these differences make the results of our referral registry unique also in a “real-world” perspective.

With the increasing numbers of RCTs comparing CABG and PCI, meta-analyses including more than 11,000 patients detected differences in clinical hard endpoints [35–37]. In 4,478 patients with ULMCAD, 5-year all-cause mortality was similar in PCI and CABG arms (10.7% vs. 10.5%) [34]. The recently published meta-analysis of Ahmad et al., including the long-term follow-up of NOBLE, EXCEL, and SYNTAXES [34], showed similar rate of overall and cardiac mortality, nonfatal myocardial infarction, and stroke with the two revascularization strategies. Moreover, D'Ascenzo et al. in their meta-analysis reported no significant difference in all-cause and cardiovascular death between PCI and CABG, although the result was mainly driven by studies using first-generation DES whereas latest RCTs using last-generation DES showed a borderline significant lower risk of global mortality with CABG [38].

A further meta-analysis by Ahn et al. showed that a complete coronary revascularization by PCI or CABG provided similar survival rates both in patients with ULMCAD and in those with high anatomical coronary complexity (SYNTAX score >32) [37]; these findings were consistent across subgroups with diabetes and multivessel disease. Hence, the high rate of complete coronary revascularization in PCI and CABG in our study might explain the similar clinical outcomes in patients with high coronary complexity. Therefore, according to our data, the ability to achieve a complete coronary revascularization, even in high coronary complex anatomy, should be the cornerstone of the clinical decision making algorithm for a “tailored” patient treatment in an era of individualized medicine.

Findings of our registry support that when contemporary revascularization strategies are adopted in the clinical management of patients with ULMCAD, the only variables independently associated with the composite endpoint are EuroSCORE and diabetes. This concept is further strengthened by the fact that we did not find any interaction between revascularization strategy by PCI or CABG and EuroSCORE or complex coronary anatomy graded by the SYNTAX score. The high predictive and independent value of EuroSCORE in ULMCAD has been already reported in a previous work [39]. In addition, consistent with our results, a recent subgroup analysis of 554 diabetics enrolled in the EXCEL trial [40] showed that diabetes is an independent predictor of the composite of death, stroke, or MI after both PCI and CABG at 3 years.

In our registry, female gender, regardless of revascularization strategy, did not influence the clinical outcome at 4 years, confirming the results of the EXCEL trial posthoc analysis [41], in which sex was not associated to adverse outcomes after ULMCAD revascularization.

Our study must be evaluated in the light of several limitations. First, the observational, retrospective design precludes causal inferences. Despite the use of multivariable analysis, it remains unknown whether residual confounders may have affected our outcome. The high number of predictors screened might have resulted in overfitting. The propensity score-adjusted analyses should have reduced it; nevertheless, given the nature of the study, residual confounders cannot be excluded. Focusing our analysis on contemporary PCI or CABG strategies has precluded the exploration of much wider patient cohorts, which could have allowed a much longer follow-up. However, we are convinced that, despite the shortcomings that are inherent to all registries, the present study provides original and clinically valuable insights into the outcomes of interventional or surgical revascularization for LMCAD in a real-world perspective.

## 5. Conclusion

In conclusion, the use of contemporary strategies aimed to obtain a state-of-the-art myocardial revascularization by PCI or CABG achieve similar outcomes, even in high-risk patients or complex coronary anatomy with ULMCAD. The clinical decision making process to choose the best management strategy for each individual patient, should take into account all the clinical characteristics, including functional and performance status, anatomic and procedural complexity, and not a merely high SYNTAX score as the main driver.

## Figures and Tables

**Table 1:** Baseline characteristics.

	All (*n* = 558)	PCI (*n* = 288)	CABG (*n* = 270)	*p* value
Age, years	71 ± 9	72 ± 10	71 ± 8	0.487
>75 years	242 (43%)	72 ± 10	114 (42%)	0.597
Male gender	451 (81%)	228 (80%)	223 (83%)	0.387
Diabetes	162 (29%)	69 (24%)	93 (34%)	0.011
Hypertension	424 (75%)	210 (73%)	214 (79%)	0.079
Dyslipidemia	349 (62%)	172 (60%)	177 (66%)	0.154
Smoker	94 (17%)	49 (17%)	45 (17%)	0.869
Previous MI	156 (28%)	77 (26%)	79 (29%)	0.507
Previous CABG	4 (1%)	2 (1%)	2 (1%)	0.948
Renal failure	93 (17%)	42 (15%)	51 (19%)	0.173
ACS	313 (56%)	157 (54%)	156 (58%)	0.438
STEMI	32 (6%)	28 (10%)	4 (2%)	<0.001
NSTEMI	228 (41%)	117 (41%)	111 (41%)	0.907
LVEF	50 ± 12	47 ± 13	52 ± 10	<0.001
LVEF ≤0.40	119 (21%)	82 (28%)	37 (14%)	<0.001
EuroSCORE	11 ± 8	10 ± 8	12 ± 7	0.028
EuroSCORE >13	11 ± 8	71 (24%)	88 (33%)	0.038

ACS, acute coronary syndrome; CABG, coronary artery bypass grafting; LVEF, left ventricular ejection fraction; MI, myocardial infraction; NSTEMI, non ST-segment elevation myocardial infarction; PCI, percutaneous coronary intervention; STEMI, ST-segment elevation myocardial infarction.

**Table 2:** Angiographic and procedural characteristics.

	PCI (*n* = 288)	CABG (*n* = 270)	*p* value
Distal LM	272 (95%)	228 (84%)	<0.001
Three-vessel disease	90 (31%)	154 (57%)	<0.001
CTO	75 (26%)	73 (27%)	0.790
RCA CTO	48 (17%)	58 (21%)	0.147
SYNTAX score >32	123 (43%)	159 (59%)	<0.001
Rotational atherectomy	21 (7.3%)	—	
IVUS	217 (76%)	—	
LM mean stent diameter (mm)	3.9 ± 0.3	—	
LM mean stent length (mm)	26 ± 12	—	
Double-stent technique	151 (52%)	—	
Crush/mini-crush	104 (69%)	—	
T-stent	25 (17%)	—	
Number of stents implanted per patient at index procedure	2.7 ± 0.9	—	
IABP	30 (10%)	—	
Max inflation pressure (atm)	21 ± 3	—	
GP IIb/IIIa inhibitors	66 (23%)	—	
Multivessel PCI	263 (91%)	—	
Successful CTO PCI	60/70 (86%)	—	
CABG beating heart	—	218 (81%)	
BIMA	—	157 (58%)	
Mean venous graft	—	0.8 ± 0.7	—
Complete revascularization	233 (81%)	233 (86%)	0.086
Mean hospital stay (days)	4.7 ± 3	10.3 ± 5	<0.001

BIMA, bilateral internal mammary artery; CABG, coronary artery bypass grafting; CTO, chronic total occlusion; IABP, intra-aortic balloon pump; IVUS, intravascular ultrasound; LM, left main; PCI, percutaneous coronary intervention; RCA, right coronary artery.

**Table 3:** Clinical outcomes.

	PCI (*n* = 288)	CABG (*n* = 270)	*p* value
*Two-year outcome*			
Primary endpoint	29 (10%)	26 (9.6%)	0.862
All-cause death	23 (7.9%)	23 (8.5%)	0.819
Cardiac death	15 (5.2%)	15 (5.5%)	0.856
Spontaneous MI	4 (1.4%)	2 (0.7%)	0.458
Stroke	2 (0.6%)	4^*∗*^ (1.4%)	0.641
Ischemia-driven revascularization	17 (5.9%)	5 (1.8%)	0.010

*Long-term outcome*	*PCI*	*CABG*	*Total p value*
Death, MI, stroke rate estimation^†^	(*n* = 288)	(*n* = 270)	0.585
1 year	5.6% ± 1.3%	7.8% ± 1.6%	
2 years	9.4% ± 1.7%	9.6% ± 1.8%	
3 years	13.3% ± 2.3%	13.7% ± 2.2%	
4 years	13.7% ± 2.2%	17.1% ± 2.6%	

^*∗*^2 patients with stroke in the surgical group died within 2 years. † Kaplan–Meier estimate. CABG, coronary artery bypass grafting; MI, myocardial infarction; PCI, percutaneous coronary intervention.

**Table 4:** Baseline and procedural characteristics of the matched population.

	PCI (*n* = 202)	CABG (*n* = 202)	*p* value
Age, years	72 ± 10	71 ± 9	0.463
Age >75 years	93 (46%)	80 (40%)	0.191
Male gender	159 (79%)	167 (83%)	0.362
Diabetes mellitus	53 (26%)	52 (26%)	0.863
Hypertension	135 (67%)	147 (73%)	0.193
Dyslipidemia	123 (61%)	132 (65%)	0.353
Previous MI	55 (27%)	57 (28%)	0.824
Renal failure	32 (16%)	57 (28%)	0.677
ACS	102 (50%)	101 (50%)	0.921
NSTEMI	82 (41%)	72 (36%)	0.306
LVEF	50 ± 11	52 ± 11	0.111
LVEF ≤0.40	37 (18%)	37 (18%)	0.999
EuroSCORE	9.7 ± 1.2	10.7 ± 1.2	0.414
EuroSCORE >13	44 (22%)	45 (22%)	0.904
Three-vessel disease + LM	116 (57%)	115 (57%)	0.920
SYNTAX score >32	106 (52%)	105 (52%)	0.921
Complete revascularization	162 (80%)	178 (88%)	0.029
Mean hospital stay, days	5 ± 3	10 ± 5	<0.001

ACS, acute coronary syndrome; CABG, coronary artery bypass grafting; LVEF, left ventricular ejection fraction; MI, myocardial infraction; NSTEM I, non ST-segment elevation myocardial infarction; PCI, percutaneous coronary intervention; STEMI, ST-segment elevation myocardial infarction.

## Data Availability

The data used to support the findings of this study are available from the corresponding author upon request.
